# Reasons given by mothers for discontinuing breastfeeding in Iran

**DOI:** 10.1186/1746-4358-7-7

**Published:** 2012-05-06

**Authors:** Beheshteh Olang, Abtin Heidarzadeh, Birgitta Strandvik, Agneta Yngve

**Affiliations:** 1 Unit for Public Health Nutrition, Department of Biosciences and Nutrition, Karolinska Institutet, Stockholm, Sweden; 2 Department of Health, Nutrition and Management, Oslo and Akershus University College of Applied Sciences, Oslo, Norway; 3 Kermanshah University of Medical Sciences, Kermanshah, Iran; 4 Breastfeeding Research Center, Tehran University of Medical Sciences, Tehran, Iran; 5 Guilan University of Medical Sciences, Rasht, Iran

**Keywords:** Family, Doctor advice, Pacifier, Infant feeding

## Abstract

**Background:**

We have previously shown that in Iran, only 28% of infants were exclusively breastfed at six months, despite a high prevalence of breastfeeding at two years of age. The primary aim of this study was to investigate the reasons women discontinued exclusive breastfeeding.

**Method:**

This retrospective study was based on questionnaires and interviews with 63,071 mothers of infants up to 24 months of age, divided into two populations: infants younger than six months and six months or older. The data were collected in 2005–2006 from all 30 provinces of Iran.

**Results:**

Only 5.3% of infants less than six months of age stopped breastfeeding (mean age of 3.2 months); more commonly in urban than rural areas. The most frequently cited reasons mothers gave for discontinuing exclusive breastfeeding were physicians’ recommendation (54%) and insufficient breast milk (self-perceived or true, 28%). Breastfeeding was common after six months of age: only 11% of infants discontinued breastfeeding, at a mean of 13.8 months. The most common reason for discontinuation at this age was insufficient breast milk (self-perceived or true, 45%). Maternal illness or medication (10%), infant illness (6%), and return to work (3%) were uncommon causes. Use of a pacifier was correlated with breastfeeding discontinuation. Maternal age and education was not associated with duration of breastfeeding. Multivariate analysis showed that using a pacifier and formula or other bottle feeding increased the risk of early cessation of breastfeeding.

**Conclusions:**

Physicians and other health professionals have an important role to play in encouraging and supporting mothers to maintain breastfeeding.

## Background

Breastfeeding provides ideal nourishment for the growth and development of infants and has a unique biological and emotional influence on the health of both mother and child [1,2]. The World Health Organization (WHO) has recommended that exclusive breastfeeding, defined as giving breast milk without any food or liquid until six months of age, confers benefits to mothers and infants [3].

We have previously shown that in 2006, only 57% of Iranian babies were exclusively breastfed at four, and 28% at six, months of age [4]. These figures were low in comparison to the WHO recommendation. However, the duration of breastfeeding was long; 57% of infants were continuing to breastfeed at two years of age [4].

This paper was written with the aim of identifying why exclusive breastfeeding at six months was low when any breastfeeding at 24 months was common. The data were obtained from the Integrated Monitoring Evaluation System Survey (IMES), which collected information constituting the basis for our previous report about the prevalence of breastfeeding in Iran [4]. In this paper we concentrate on geographic differences, including urban/rural location, as well as reasons given by mothers for stopping breastfeeding.

## Methods

### Definitions

We used the definition of breastfeeding given by WHO [3,5]. *Exclusive breastfeeding* was defined as only breast milk given to the infants and no other liquids or solids except for those containing vitamins or medicines; *partial breastfeeding* included addition of some artificial feeds, either milk or cereal, or other food; *any breastfeeding* including partial and exclusive breastfeeding. *Discontinuation* is defined as complete cessation of any breastfeeding [6] and *early discontinuation* as cessation before six months [6].

### Data collection

The collection of data on breastfeeding was made by health workers in the IMES from 15 September 2005 to 15 January 2006, as previously reported [4]. The study population included all infants less than 24 months of age who lived in urban or rural areas and had been referred to a Health Care Center to receive health care, vaccination or treatment for any illness during the period 15 September 2005 to 15 January 2006 (in rural areas through home visits by trained interviewers, who were employees of the Ministry of Health (MOH)) [7]. Data about discontinuation of breastfeeding before 6 months of age included 8,434 infants. For any breastfeeding after 6 months of age, 52,637 infants were studied.

Data collection was performed by interviewing mothers, using a preprinted questionnaire format. The questionnaire used was validated in a pilot project in IMES in 2004. Results from this pilot study were used for calculating sample size [4]. The questionnaire included questions about the name of province, district and Health Care Center; numbers in household, mother’s age and education, infant’s age, infant feeding pattern during the last 24 hours. Breastfeeding was reported as no breastfeeding or any breastfeeding and before 6 months of age also as exclusive breastfeeding. Breast milk substitutes or other drinks in bottles (sweetened water, tea, juice, etc) were registered as well as the use of a pacifier.

### Statistical analysis

IMES data were analyzed using Stata version 8.0 and Survey Analysis commands by using each medical university as a separate stratum and each data collection area as primary sampling unit (PSU) and the proportion of sampled persons to the population of under two years of age as weight. The rates of discontinuation in different age groups were calculated with 95% confidence interval (CI). Multivariate analysis using Stata 8.0 and XT logistic regression models in survey analysis mode were used. The same PSUs were used for analyzing association between discontinuation rate of breastfeeding and background variables.

### Ethical approval

Approval to conduct this analysis was obtained from the Ethics Committee at the MOH in Iran in February 2007. Informed consent was obtained from mothers before the interview. Ethics approval for the analyses of the results to be conducted in Sweden was obtained in 2009 from the Regional Ethical Review Board at Karolinska Institutet.

## Results

### Infants less than 6 months of age (n = 8,434)

**Figure 1:**
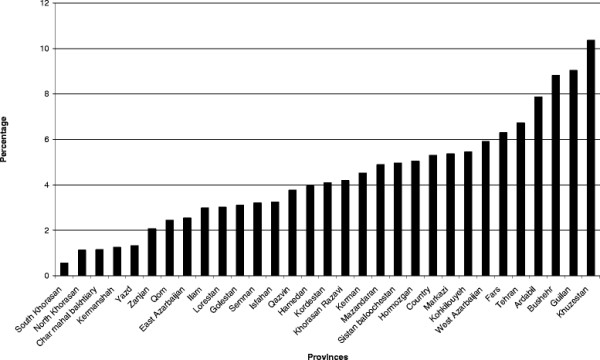
**The percentage of discontinuation of breastfeeding before 6 months.** Data from IMES.

**Figure 2:**
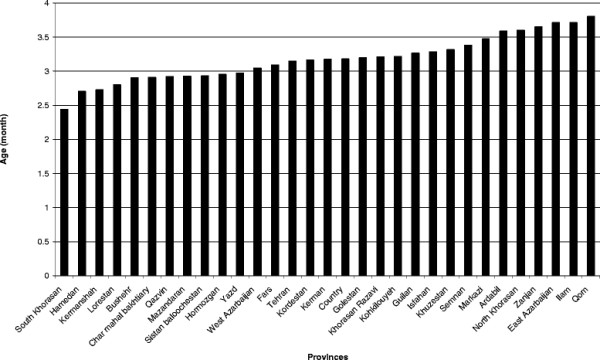
**Mean age in months of discontinuation of breastfeeding before 6 months.** Data from IMES.

The mean age of the mother in different regions ranged from 24.2 to 29.0 years. The percentage of early discontinuation of breastfeeding (before 6 months) was 5.3% (Figure 1). The mean age of infant at early discontinuation of breastfeeding in the country was 3.2 months, ranging from 2.4 to 3.8 months in the different regions (Figure 2). At the age of six months, only 27.7% of the infants were exclusively breastfed.

Reasons for early discontinuation of *exclusive* breastfeeding during the first six months of life (72.3%) are given in Table 1. The main reason given by the mother was that discontinuation was advised by the physician (54%). There was no difference between urban and rural areas.

**Table 1:** Percentage distributions of reasons for discontinuing exclusive breastfeeding before six months of age in 8,434 infants in Iran

Reasons for discontinuing exclusive breastfeeding*	**Mean (95% CI)**
Physician’s recommendation	54 (53, 55)
Insufficient breast milk	28 (27, 29)
Family recommendation	20 (19, 21)
Crying baby	17(16, 18)
Non-specific reason	17(16, 18)

*Mothers could give more than one reason.

Multivariate analysis and XT logistic regression models showed that living in urban areas (adjusted Odds Ratio [aOR] = 1.3, 95% CI, 1.1, 4.60), using a pacifier (aOR = 2.4, 95%CI, 2.0, 4.6), formula or other bottle feeding (aOR = 16.8, 95%CI, 8.1, 26.0) increased the risk of early breastfeeding discontinuation significantly (Table 2). Maternal age and education and numbers in the household were not significantly correlated with breastfeeding discontinuation in the first six months.

**Table 2:** Multivariate analysis for discontinuing breastfeeding in two population groups (< and ≥ 6 months of age)

**Variable**	**<6 months of age Adjusted Odds Ratio (95% CI)**	**≥6 months of age Adjusted Odds Ratio (95% CI)**
Living in urban areas (Reference group: rural areas)	1.3 (1.1, 4.60)	1.1 (0.88, 1.57)
Return to work outside of home (Reference group: mother did not work outside the home)	16 (7.45, 30.65)	1.05 (1.0, 3.87)
Age of infant (Reference group: infant < 1 month)	1.2 (1.08, 2.63)	1.2 (1.10, 1.69)
Using a pacifier (Reference group: did not use a pacifier)	2.4 (2.0, 4.6)	2.6 (1.38, 4.19)
Using formula or other bottle feeding (Reference group: did not bottle feed)	16.6 (8.1, 26.0)	12 (5.02, 20.84)
Numbers in household (Reference group: ≥ 3 persons: mother, father and one child)	1.12 (0.88, 1.43)	1.00 (0.88, 1.13)
Mother’s age (Reference group: < 19 years)	1.03 (0.98, 1.09)	0.99 (0.96, 1.03)
Mother’s education (Reference group: illiterate)	1.54 (0.95, 2.50)	0.93 (0.72, 1.21)

*Adjusted for all variables in the table.

### Infants over 6 months of age (n = 52,637)

**Figure 3:**
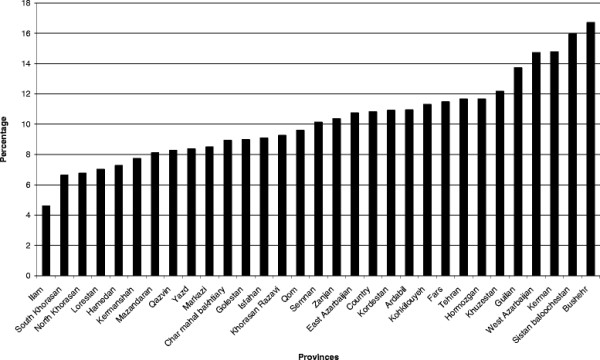
**The percentage of discontinuation of breastfeeding after 6 months of age.** Data from IMES.

**Figure 4:**
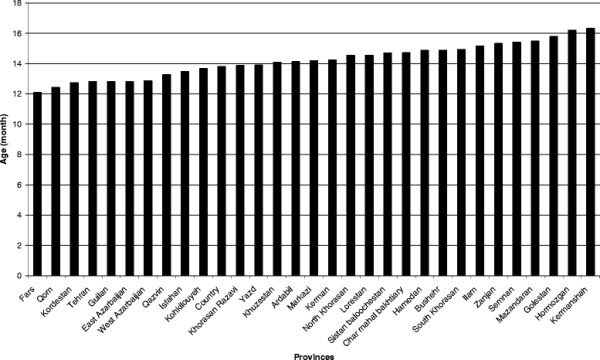
**Mean age in months of discontinuation of breastfeeding after 6 months of age.** Data from IMES.

The mean age of the mothers ranged from 25.7 to 29.4 years in the different regions. Discontinuation of breastfeeding after six months was identified in 11% of the mothers (Figure 3), with a mean infant age of 13.8 months (Figure 4). The reason given by mothers for discontinuation of any breastfeeding in infants after 6 months of age was most often insufficient breast milk (real or perceived) (Table 3). Return to work outside the home was seldom stated as a reason for discontinuing breastfeeding.

**Table 3:** Percentage distributions of reasons for discontinuing breastfeeding after 6 months of age in 52,637 infants in Iran

**Reasons for discontinuation of breastfeeding ≥6 months of age**	**Mean (95% CI)**
Insufficient breast milk	45 (43, 47)
Maternal illness or medication	11 (10, 12)
Return to work	3 (2, 4)
Time to stop	6 (5, 7)
Breast refusal	27 (26, 29)
Infant illness	6 (5, 7)
Non-specific reason	25 (24, 27)

*Mothers could give more than one reason.

Multivariate analysis and XT logistic regression models demonstrated that, using a pacifier (aOR = 2.6, 95% CI, 1.38, 4.19) and formula or other bottle feeding (aOR = 12, 95% CI, 5.02, 20.84), were positively correlated with breastfeeding discontinuation (Table 2). Maternal age and education and number in the household were not associated with breastfeeding discontinuation.

## Discussion

Our findings indicated that the rate of early discontinuation of breastfeeding (before six months of age) was low (5.3%) in Iran, and discontinuation was also relatively low after six months of age (11%). However, the prevalence of exclusive breastfeeding at six months was only 28%. The most common reason for discontinuation of exclusive breastfeeding was, according to the mother, advice from the physician. In Iran, pediatricians, obstetricians and general physicians are responsible for consultations about breastfeeding continuation. The physicians give advice in health centers and the private sector. We don’t know if mothers received recommendations in the public or private sectors [8]. It can not be excluded that mothers reported that advice was given by physicians or health professionals even though it was the mother’s own decision, as this might be more socially acceptable than saying they decided themselves.

Taveras et al. reported in multicultural study groups that mothers will not usually discontinue breastfeeding within the first three months after delivery if they are encouraged by physicians to continue [9]. The American Academy of Pediatricians recommends physicians to advise mothers on initiation and continuation of breastfeeding and to be knowledgeable about the basics of lactation [10]. It was thus surprising that doctors’ recommendations were among the most important reasons to stop exclusive breastfeeding, since the physiological and psychological beneficial effects of breastfeeding should be well acknowledged by them. Similar influences by physicians have been reported in a study in Turkey [11]. A study in Puerto Rico showed that as many as 26% of primary care physicians did not encourage exclusive breastfeeding [12]. A study in France showed that trained primary care physicians were effective in increasing breastfeeding duration [13].

The most common reason given for discontinuation of any breastfeeding was insufficient breast milk. A previous study from Tehran showed that 74% of mothers started to provide complementary feeding to their infants due to insufficient breast milk [14]. It should be questioned if Iranian mothers really have insufficient breast milk production. Breast refusal was reported as a common reason for discontinuation of breastfeeding in infants after 6 months of age, and might be related to insufficient milk supply, psychological factors or infant’s factors [15]. The reasons given by the mothers before 6 months of age may be more related to maternal factors, while the reasons given by mothers to older infants may be related to infants.

Maternal smoking is usually associated with shorter duration of breastfeeding [16], but data on smoking was not collected in this study.

Family recommendation constituted 20% of causes that influenced mothers to stop exclusive breastfeeding. The support of the family has also been stressed by others [17], and the lack of support was shown in one study to be one of the reasons for mothers to stop early breastfeeding [11]. In line with this, others have reported that mothers identified social support as more important than health service support [18].

The introduction of pacifiers has been shown to affect breastfeeding duration negatively [19], which was corroborated in our study. While maternal age has been shown to have a positive correlation with duration of breastfeeding [19,20], we did not find this.

Access to early breastfeeding after delivery has been shown to influence the duration of both exclusive and any breastfeeding positively [21]. Early access is practiced in Iran and would at least be consistent with the long maintenance of any breastfeeding reported in our study.

Factors positively influencing the extent of breastfeeding have been shown to be mothers’ sociodemographic and psychosocial background, such as high education in the mothers and a supportive family [22]. In contrast, it was reported in Iran that higher maternal education had a negative influence, as well as a high paternal income [14,23].

A limitation of the study is the retrospective approach and using collected data from many different Health Care Centers (current status data). However, data collection was performed using identical instructions and protocols. Furthermore, 23% of the mothers did not specify the reasons for the cessation of breastfeeding. Although some mothers may have stopped just after the survey or continued until 24 months. Our results are supported by other findings in stressing the importance of support from family and especially from physicians [24].

## Conclusion

The cause of early discontinuation of exclusive breastfeeding during infancy, as given by the mother, was mainly physicians’ recommendation and/or insufficient breast milk. Specific reasons, such as maternal or infant illness were rare, indicating that by support and education, the prevalence of exclusive breastfeeding to six months could be increased. The results suggest an important role for physicians and other health professionals to encourage breastfeeding.

## Competing interests

The authors declare that they have no competing interests.

## Authors’ contributions

BO drafted the manuscript and coordinated the project between the MOH in Iran and Karolinska Institutet in Sweden. AB was responsible for IMES data and performed statistical analysis. BS helped to developing the study and writing the manuscript. AY participated in design of the study. All authors approved the final manuscript.
